# Molecular mechanisms underlying uremic toxin-related systemic disorders in chronic kidney disease: focused on β_2_-microglobulin-related amyloidosis and indoxyl sulfate-induced atherosclerosis—Oshima Award Address 2016

**DOI:** 10.1007/s10157-018-1588-9

**Published:** 2018-06-05

**Authors:** Suguru Yamamoto

**Affiliations:** Division of Clinical Nephrology and Rheumatology, Niigata University Graduate School of Medical and Dental Sciences, 1-757 Asahimachi-dori, Niigata, 951-8510 Japan

**Keywords:** Uremic toxins, β_2_-Microglobulin, Dialysis-related amyloidosis, Indoxyl sulfate, Atherosclerosis, Macrophages

## Abstract

Uremic toxins are linked to chronic kidney disease (CKD)-related systemic diseases. β_2_-Microglobulin (β_2_-m), a water-soluble, middle-sized molecule, is associated with mortality and dialysis-related amyloidosis (DRA). DRA occurs in long-term dialysis patients, with β_2_-m amyloid deposited mainly in osteoarticular tissues. We investigated a model of β_2_-m amyloid fibril extension at neutral pH in the presence of trifluoroethanol or sodium dodecyl sulfate. Using this model, some biological molecules, including glycosaminoglycans and lysophospholipids, were found to be chaperones for β_2_-m amyloid fibril extension. Several protein-bound solutes, such as indoxyl sulfate (IS) and *p*-cresyl sulfate, are independent risk factors for cardiovascular disease in CKD patients, especially those undergoing dialysis. We investigated kidney injury-induced acceleration of atherosclerosis in association with macrophage phenotypic change to a proinflammatory state as well as increased IS deposition in lesions in an animal model. IS directly induced macrophage inflammation and impaired cholesterol efflux to high-density lipoprotein (HDL) in vitro. In addition, a clinical study showed that HDL isolated from CKD patients induced proinflammatory reactions and impaired cholesterol efflux to macrophages. These findings suggest that protein-bound solutes, including IS, will induce dysfunction of both macrophages and HDL in atherosclerotic lesions. To remove uremic toxins efficiently, we demonstrated the potential efficacy of oral charcoal adsorbent and hexadecyl-immobilized cellulose beads in hemodialysis patients. These findings suggest that uremic toxins induce various CKD-related systemic disorders, and further therapeutic strategies will be needed to reduce uremic toxins enough and improve life expectancy in CKD patients.

## Uremic toxins and systemic disease in chronic kidney disease patients

Advanced chronic kidney disease (CKD) induces various systemic diseases including cardiovascular disease (CVD), osteoarticular disorders, infections, malignant disease, and others. The frequency and severity are enhanced with the progression of CKD, especially end-stage kidney disease with dialysis treatment [1]. CKD-related systemic disease not only worsens survival, but also impairs activities of daily living (ADL) and quality of life (QOL). Thus, greater understanding of the mechanism of these disorders and investigation of therapeutic strategies is necessary. An accumulation of uremic toxins is a CKD-specific factor in the development of CKD-related systemic disease. Despite recent progress in dialysis treatment and the preservation of kidney function [2], survival and ADL/QOL in CKD patients have not improved enough.

My collaborators and I have studied the pathophysiology of uremic toxin-related systemic disorders, especially dialysis-related amyloidosis (DRA) and atherosclerosis, with a focus on β_2_-microglobulin (β_2_-m) and indoxyl sulfate (IS), respectively, and tried to identify therapeutic strategies to improve survival and ADL/QOL in CKD patients.

Progressive kidney disease induces uremic syndrome, with the retention of various solutes that are normally excreted by the kidney. Solutes with biological toxicity, direct or indirect, are called “uremic toxins.”

Requirements for a uremic toxin are include the following [3–5]:

1. The toxin is a unique chemical entity.
2. Quantitative analysis of the toxin in biological fluids is possible.
3. The levels of the toxin in biological fluids increase with deterioration of kidney function.
4. A positive relationship between toxin level in biological fluids and manifestations of uremic syndrome is present.
5. Administration of the toxin at a concentration seen in patients with kidney disease shows toxic effects related to uremic syndrome, both in vivo and in vitro.

A literature search identified 88 uremic toxins in 621 articles. These were classified into groups according to molecular weight and protein-bound properties, and were water-soluble low molecular weight, middle sized, and protein-bound molecules [3].

## β_2_-Microglobulin and dialysis-related amyloidosis

**Fig. 1:**
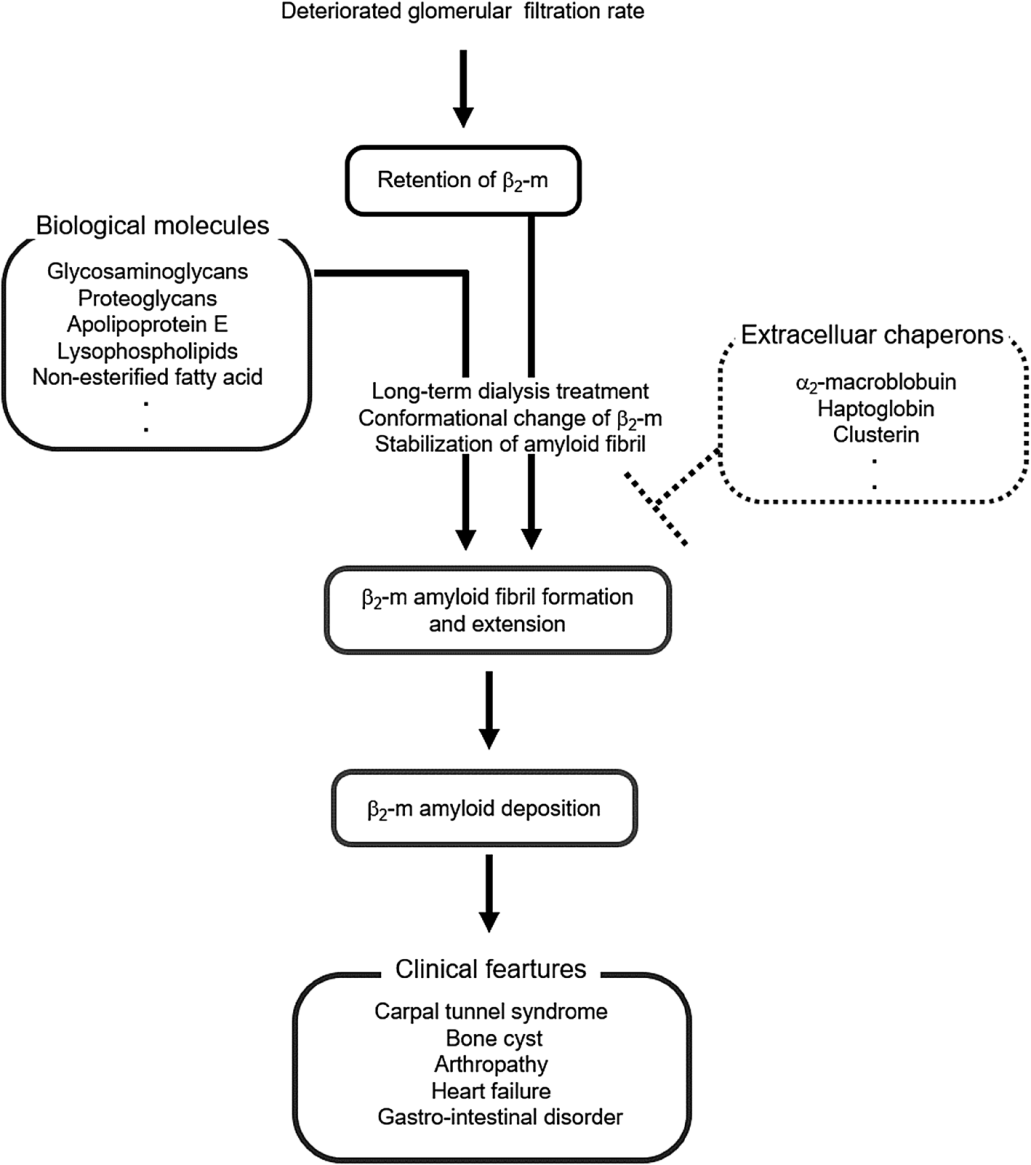
Pathogenesis of dialysis-related amyloidosis. β_2_-Microglobulin (β_2_-m), a water-soluble, middle sized uremic toxin, increases with the deterioration of kidney function. Some biological molecules, such as glycosaminoglycans and proteoglycans, change the conformation of β_2_-m and stabilize and extend the amyloid fibrils. In contrast, extracellular chaperones including α_2_-macroglobulin may inhibit amyloid fibril formation by capturing unfolded and misfolded β_2_-m

A representative, water-soluble middle sized molecule, β_2_-m (11.8 kDa), is associated with survival in dialysis patients [6–8]. For example, the randomized Hemodialysis (HEMO) Study showed that predialysis serum β_2_-m levels were associated with all-cause mortality [8], as well as mortality owing to infections in dialysis patients [9]. In CKD-related osteoarticular disorders, β_2_-m is a precursor protein for DRA [10]. β_2_-m-related amyloid fibrils are formed and deposited primarily in osteoarticular joint tissues, resulting in various osteoarticular disorders, such as carpal tunnel syndrome, destructive spondyloarthropathy, and bone cysts in dialysis patients [11]. Accumulation of β_2_-m and the interactions between β_2_-m and other biological molecules are thought to be needed for amyloid fibril formation in vivo [12, 13]. The β_2_-m-related amyloid fibril formation and extension occurs according to a nucleation-dependent polymerization model [12, 14]. This model consists of a nucleation phase and an extension phase. Nucleus formation requires a series of monomer association steps, which represent the rate-limiting step in amyloid fibril formation. Once the nucleus (*n*-mer) has been formed, further addition of monomers becomes thermodynamically favorable, resulting in the rapid extension of amyloid fibrils according to a first-order kinetic model [12, 14]. In the mechanism of amyloidogenesis of natively folded proteins as well as β_2_-m, partial unfolding is believed to be a prerequisite to assembly into amyloid fibrils, both in vitro and in vivo. In this process, conformational change of β_2_-m with biological molecules is necessary [12, 15]. The extension of β_2_-m-related amyloid fibrils, as well as the formation of the fibrils from β_2_-m, is greatly dependent on the pH of the reaction mixture, with the optimum pH being around 2.0–3.0 [15, 16]. On the other hand, the fibrils readily depolymerize into monomeric β_2_-m at pH 7.5 [17]. Thus, to observe the extension of β_2_-m-related amyloid fibrils at neutral pH, we need to unfold the compact structure of β_2_-m monomer to an amyloidogenic conformer, and stabilize the extended fibrils by adding other factors. We investigated the effect of low concentrations of 2,2,2-trifluoroethanol (TFE) and sodium dodecyl sulfate (SDS) on the extension of β_2_-m-related amyloid fibrils at neutral pH in vitro [18, 19]. TFE at concentrations of up to 20% (v/v) or SDS at a critical micelle concentration caused amyloid fibril extension by inducing a subtle change in the tertiary structure of β_2_-m, and stabilizing the fibrils at neutral pH. TFE-induced amyloid fibril extension at neutral pH was enhanced by several kinds of glycosaminoglycans, especially heparin [18]. In these reactions, glycosaminoglycans bound directly to the amyloid fibrils. In another study, depolymerization of amyloid fibrils at pH 7.5 was inhibited dose-dependently by the presence of apolipoprotein E, some glycosaminoglycans, or proteoglycans [17, 20]. The results suggested that those biological molecules could enhance the deposition of β_2_-m-related amyloid fibrils in vivo, possibly by binding directly to the surface of the fibrils and stabilizing the conformation of β_2_-m in the fibrils [12]. Using an in vitro β_2_-m amyloid fibril formation model, other studies showed that several other biological molecules including lysophospholipids [21] and various non-esterified fatty acids [22] are enhancing-factor candidates for β_2_-m-related amyloid fibril deposition in vivo. Thus, deposition of β_2_-m-related amyloid requires β_2_-m conformational change and stabilization of amyloid fibrils with some biological molecules (Fig. 1). In contrast, recent findings showed that extracellular chaperones including α_2_-macroglobulin may inhibit amyloid fibril formation by capturing unfolded and misfolded β_2_-m [23]. Further clinical studies will be needed to verify the in vivo roles of these molecules in DRA. The β_2_-m-related amyloid fibrils deposited in tissues induce cellular interactions that are associated with DRA symptoms, such as carpal tunnel syndrome and destructive spondyloarthropathy. When synovial fibroblast cells were reacted with extended β_2_-m-related amyloid fibrils in vitro, cellular survival were impaired by disrupting endosomal/lysosomal membranes [24]. This reaction may be associated with the development of carpal tunnel syndrome in CKD patients. Macrophages in spine lesions are thought to be activated by deposited amyloid fibrils, and activated macrophages may accelerate destruction of spine with long-term dialysis treatment [25].

## Indoxyl sulfate and atherosclerosis

**Fig. 2:**
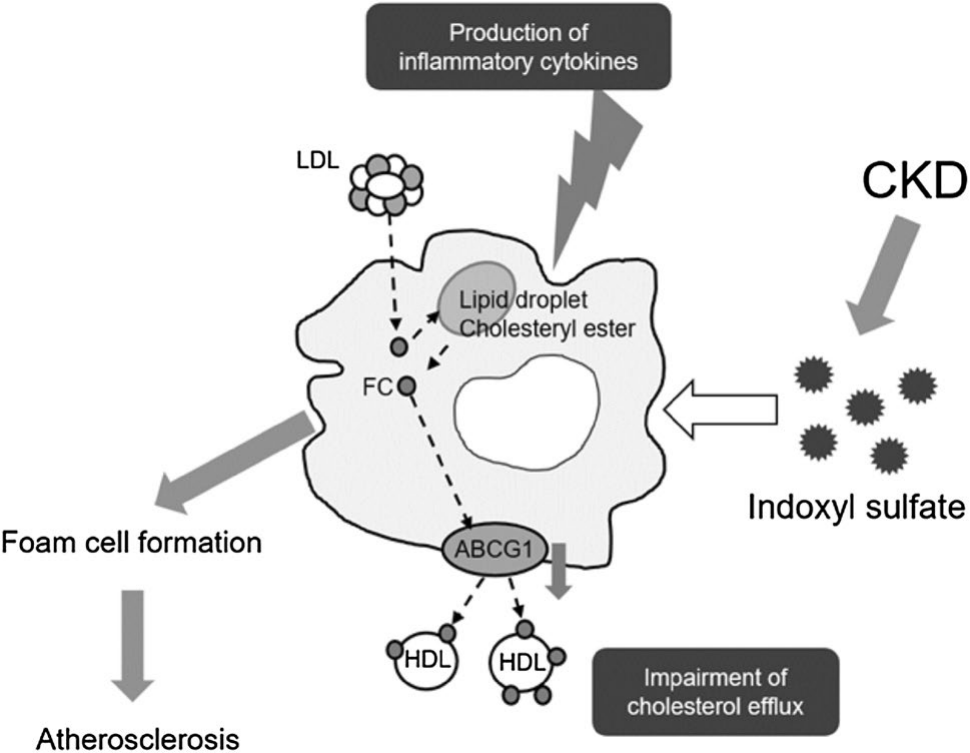
Indoxyl sulfate induces macrophage foam cell formation in atherosclerotic lesions. Indoxyl sulfate, a protein-bound uremic toxin, reacts directly with macrophages and induces production of inflammatory cytokines as well as impairment of cholesterol efflux to high-density lipoprotein, leading to macrophage foam cell formation. *ABCG1* ATP-binding cassette transporter G1, *CKD* chronic kidney disease, *FC* free cholesterol, *LDL* low-density lipoprotein, *HDL* high-density lipoprotein

CKD is one of the strongest risk factors for CVD owing to progressive atherosclerosis as well as vascular calcification. Accumulation of protein-bound uremic toxins is associated with cardiovascular mortality in CKD patients [26–28]. Serum levels of IS increase with the progression of CKD, particularly in patients undergoing dialysis. Production of indole, precursor of IS, by intestinal flora is enhanced with kidney disease setting, animal models suggested that use of Lubiprostone modulated kidney damage-induced perturbation of microbiota and reduced IS production [29, 30]. IS was associated with increased cardiovascular mortality, aortic calcification, and pulse wave velocity in CKD patients [28]. Indole acetic acid (IAA) showed trends similar to IS, and multivariate analysis showed that IAA, but not IS or *p*-cresyl sulfate, remained a significant predictor of mortality and cardiovascular events [26]. In animal models, subtotal nephrectomy accelerated atherosclerosis as well as plaque formation in apolipoprotein E knockout mice [31]. In atherosclerotic lesions, renal injury induced macrophage phenotypic change, with an increase in proinflammatory M1 as well as a decrease in anti-inflammatory M2 [32, 33]. Our research suggested that kidney injury-induced acceleration of atherosclerosis is associated with IS [31], the renin-angiotensin-aldosterone system [32], and peroxisome proliferator-activated receptor-γ [33]. These clinical and basic studies suggested that protein-bound uremic toxins, especially IS, act as major CKD-specific factors in CKD-induced acceleration of atherosclerosis. When macrophages differentiated from THP-1 cells were exposed to IS in vitro, IS decreased cell viability but promoted macrophage inflammatory cytokine production as well as reactive oxygen species production [34]. In this process, IS-inducing inflammation in macrophages results from accelerating aryl hydrocarbon receptor-NF-κΒ/MAPK cascades, but not the NLRP3 inflammasome [35]. These reactions may restrict mature IL-1β production, which may explain sustained chronic inflammation in CKD patients. IS also reduced macrophage cholesterol efflux and decreased ATP-binding cassette transporter G1 expression [34]. Thus, direct interactions of IS with macrophages induces macrophage foam cell formation, which leads to atherosclerosis acceleration in patients with CKD (Fig. 2). We also found that HDL from CKD patients but not from non-CKD subjects impaired macrophage cholesterol efflux [36]. Although HDL is known to have anti-inflammatory activity, uremic HDL enhanced macrophage inflammation as well as migration [36]. These results suggest that uremic toxins may induce functional abnormalities in macrophages and HDL that enhance macrophage foam cell formation in atherosclerotic lesions in CKD patients (Fig. 2) [37]. IS or uremic HDL also induced functional abnormalities of not only macrophages but other atherosclerosis-associated cells including endothelial cells [38, 39]. Thus, systemic removal of uremic toxins will be effective to prevent CKD-induced disorders.

## Strategies to remove uremic toxins

**Fig. 3:**
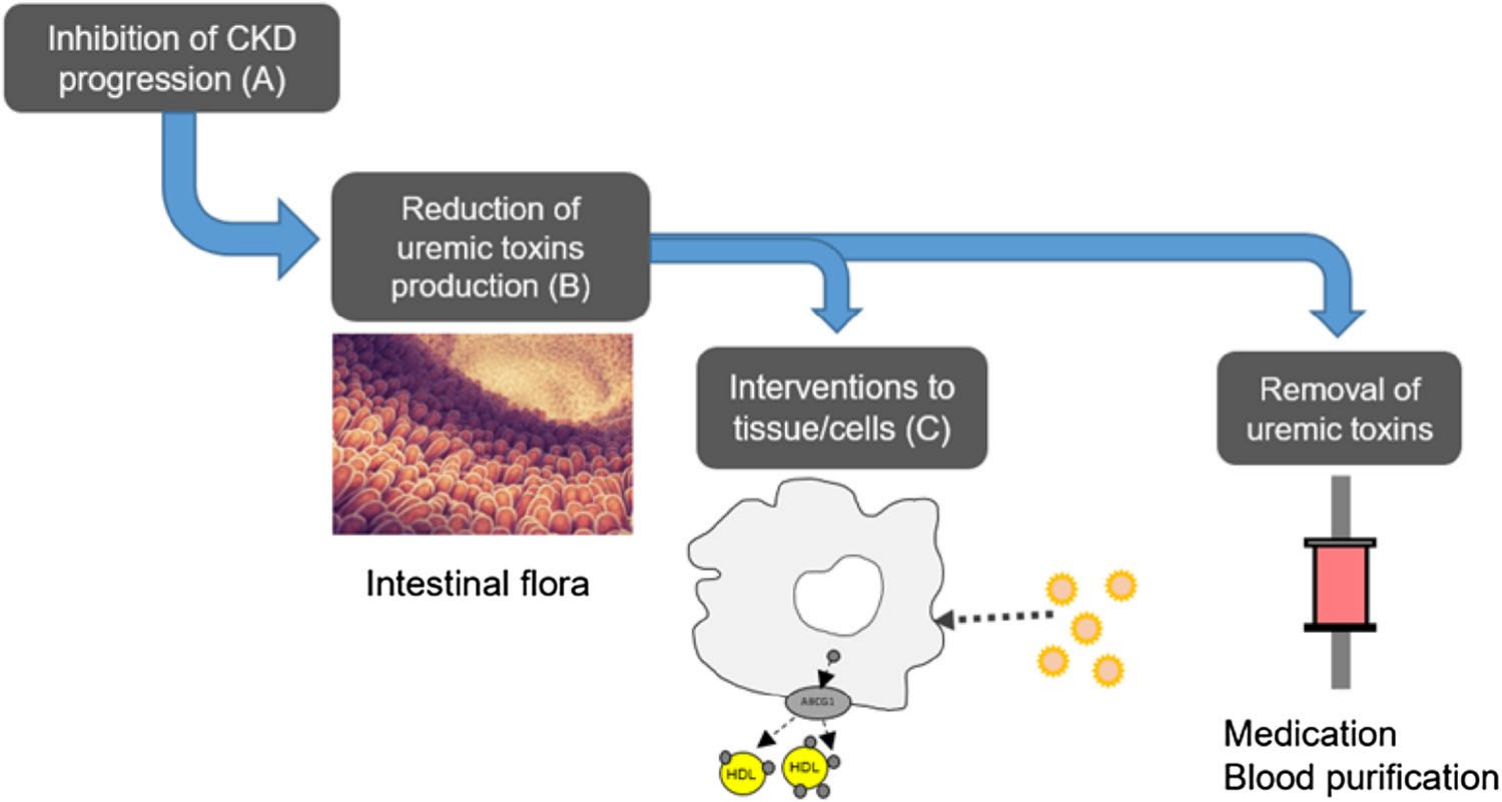
Therapeutic strategies for uremic toxin-related systemic disorders. Therapeutic strategies for the reduction of uremic toxins should include (A) preservation of kidney function, (B) inhibition of uremic toxin production, (C) prevention of the interaction between uremic toxins and tissues/cells, and (D) removal of uremic toxins with medication or blood purification therapy

To prevent CKD-related systemic disease including CVD, preservation of kidney function, including treatment for glomerulonephritis and diabetic nephropathy, is essential to avoid accumulation of uremic toxins. In advanced CKD, especially end-stage kidney disease, removal of uremic toxins with medication and blood purification therapy will be another option for the prevention of CKD-related systemic disease. An oral charcoal adsorbent (AST-120) reduces serum levels of IS [40, 41] and can be used in advanced CKD patients for the preservation of kidney function while some large interventional clinical studies did not show clear effect on it [42–46]. Reduction of uremic toxins with AST-120 may be associated with better outcomes in CKD-related systemic disease. In fact, kidney damage-induced acceleration of atherosclerosis was modulated with administration of AST-120, with less aortic deposition of IS and aortic expression of inflammatory cytokines [31]. Another study showed that AST-120 modulated CKD-induced cardiac damage, with decreased serum/urine levels of IS and oxidative stress markers, such as 8-hydroxy-2′-deoxyguanosine and acrolein, in a rat model [47]. IS strongly bound to high molecular weight protein and is difficult to remove with conventional dialysis treatment. A clinical study showed that IS in serum is 97.7% protein-bound and is only reduced by 31.8% with standard hemodialysis [4]. Recent findings showed that a longer hemodialysis treatment session [48], use of large-pore, super-flux cellulose triacetate membranes [49], and hemodiafiltration [50] increased the removal of protein-bound uremic toxins; however, these changes are thought to be insufficient to prevent CKD-related complications. Additional therapy with conventional dialysis is needed to adequately remove protein-bound uremic toxins. For example, when anuric patients undergoing maintenance hemodialysis used AST-120 6 g/day for 2 weeks, serum IS, *p*-cresyl sulfate, and phenyl sulfate levels in the predialysis session decreased significantly [51], as did oxidative stress markers including oxidized albumin and 8-isoprostane [51]. The Lixelle^®^ column contains porous hexadecyl-immobilized cellulose beads and was developed for direct hemoperfusion of blood β_2_-m with hydrophobic interactions [52, 53]. Recent research found that hexadecyl-immobilized cellulose beads adsorbed protein-unbound free IS, *p*-cresyl sulfate, phenyl sulfate, and IAA to some degree [54]. These interventions are problematic in clinical use, and further clinical investigation will be necessary to adequately reduce uremic toxins. Methods for reduction include targeting of intestinal flora that produce uremic toxins, removal of circulating uremic toxins, and others (Fig. 3). Treatments at each stage will decrease uremic toxins and prevent CKD-related systemic disorders. In addition, adequate removal of protein-bound uremic toxins should be recommended when the interventions can improve survival and ADL/QOL in CKD patients.

## Conclusion

Uremic toxins and CKD-related diseases, focused on β_2_-m-related amyloidosis and IS-induced acceleration of atherosclerosis, were reviewed, based on current knowledge and future perspectives. Accumulation of uremic toxins can induce various systemic disorders, and each uremic toxin has unique characteristics, such as conformational change and protein-binding properties in the disease setting. Further studies will be needed to identify the characteristics of each uremic toxin in greater detail and to develop therapeutic strategies for improved survival and ADL/QOL in CKD patients.
